# ‘Better Conversations with Primary Progressive Aphasia and other rare dementias (BCPPA plus)’ telehealth communication partner training programme: protocol for an NHS embedded randomised controlled feasibility and implementation study

**DOI:** 10.1136/bmjopen-2026-117509

**Published:** 2026-08-07

**Authors:** Richard Talbot, Roisin Keogh, Lola Rosenthal, Bethany Anthony, Mark Pearson, Rhiannon Tudor Edwards, Maya L Henry, Jason Warren, Rosemary Varley, BCPPA Patient and Public Involvement Advisory Group, Anna Volkmer

**Affiliations:** 1 Division of Psychology and Language Sciences, University College London, London, UK; 2 Centre for Health Economics and Medicines Evaluation (CHEME), Bangor University, Bangor, UK; 3 Wolfson Palliative Care Research Centre, Hull York Medical School, University of Hull, Hull, UK; 4 Departments of Speech, Language, and Hearing Sciences and Neurology, The University of Texas at Austin, Austin, Texas, USA; 5 Dementia Research Centre, Queen Square Institute of Neurology, University College London, London, UK

**Keywords:** Dementia, Caregivers, Rehabilitation medicine, Natural Language Processing

## Abstract

**Introduction:**

Language and communication difficulties are common across a range of dementias including primary progressive aphasia (PPA). Better Conversations with PPA (BCPPA) is a co-designed communication partner training intervention that aims to improve the experience of conversations for a person with communication difficulties. An NHS-based pilot study of face-to-face BCPPA demonstrated positive outcomes but also raised questions about when and where this intervention should be delivered (at diagnosis or later; face to face and/or remotely), how to best measure the effectiveness of the intervention in line with participant’s priorities and whether BCPPA might be acceptable and useful to people with a range of dementia types. We also had questions about the possible economic implications of BCPPA. In preparation for a future full effectiveness study, the present study will address the following questions:

**Implementation:**

What is the optimal schedule and dosage of BCPPA versus treatment as usual?

**Feasibility:**

What are the eligibility criteria for the BCPPA intervention? Is remote delivery of BCPPA acceptable to people with PPA and other dementias? Can the BCPPA be delivered remotely as intended? Do planned outcome measures show sensitivity to change pre-/post-/3 months post BCPPA? What are the barriers and facilitators to implementation of BCPPA in an NHS setting? What is the most appropriate perspective of analysis and way of measuring costs and outcomes in a future cost-effectiveness analysis of BCPPA versus usual care?

**Methods and analysis:**

In line with Medical Research Council guidance on development and management of complex interventions, Skivington *et al*, 2021 this protocol paper describes a phase II mixed-methods process evaluation and health economic study. This protocol for a randomised controlled pilot feasibility study compares the BCPPA communication partner training intervention with a deferred entry group for people with PPA and other rare dementias and their communication partners. Participants will be recruited at diagnosis and review appointments from two NHS trusts. Participants who have completed a repeated baseline measure will be randomised to either the BCPPA intervention or a deferred entry group. The intervention will be delivered remotely, via teletherapy, over 4–6 weeks depending on goal achievement. The deferred entry group will receive the intervention after a 6-week waiting period, and all participants will be assessed immediately post intervention and again at 3 months post intervention. Outcome measures have been selected in line with the current recommendations for a core outcome set for PPA and to address questions of implementation and health economic evaluation. Qualitative and quantitative analysis methods will be used to explore the data.

**Ethics and dissemination:**

Ethical approval for this study was granted by the Health Research Authority for England and Wales IRAS (Project ID: 341322 REC REF 24-NI-0123). Results from this study will be published in peer-reviewed journal articles and shared with participants and patient and public involvement advisors in accessible formats.

**Trial registration number:**

ISRCTN16268666.

**Strengths and limitations of this study**

- This study describes a randomised trial exploring the impact of communication partner training interventions for people with dementia.
- This study employs various outcome measures that were not designed for people with dementia.
- The co-produced intervention and associated research have been undertaken with guidance from people with lived experience from the very outset.

## Background

 Being able to communicate effectively is fundamental to our daily lives and well-being. When impaired, this has a significant negative impact on quality of life.^1^ Although progressive language difficulty is a common secondary symptom in many dementia types,^2^ primary progressive aphasia (PPA) describes a group of language-led dementias associated with frontotemporal dementia (FTD) or Alzheimer’s disease (AD) in which a progressive difficulty with language is the primary and dominant symptom, affecting all aspects of daily living.^3 4^ Conservatively, approximately 300 000 people are living with PPA in the UK.^5^ In contrast, the burden of AD has been well documented, accounting for 50%–75% of the estimated 990 000 people living with dementia in the UK.^6 7^ Language difficulties are a major contributor to overall disability and reduced quality of life in AD.^8 9^ However, language difficulties in dementias such as AD continue to be under-recognised and under-treated.

The impact of communication difficulties in PPA and AD ripples beyond the person and affects their social and occupational networks. Communication difficulties across all dementia types result in a breakdown in conversational exchanges, whether due to word finding, sentence formulation or more general cognitive impairments.^10^ Communication difficulties carry a high disease burden and are often the primary cause of relationship breakdown.^11^ People with PPA report increasing social isolation, and depression and anxiety are not uncommon.^1^ Spouses of people with PPA report feelings of gradual loss of relationship and meaningful social interaction, as well as the increasing dependency on them to help the person with PPA communicate.^12^ Links between relationship quality and loneliness in dementia have informed recommendations for interventions that help maintain social relationships and thereby help reduce loneliness.^13^ The National Institute for Health and Care Excellence guidelines for dementia research recommendations^14^ and the dementia research roadmap for prevention, diagnosis, intervention and care by 2025^15^ both emphasise as priorities the development and evaluation (including cost-effectiveness) of interventions for people living with dementia and their carers, particularly those focused on everyday functioning and well-being.

The main treatment for communication difficulties in PPA and AD is speech and language therapy. A range of interventions has been developed^16^ to address speech, language and communication difficulties of people with PPA and AD. However, a consensus study that asked people living in the community with dementia what they wanted from non-pharmacological interventions highlighted maintenance of communication and relationships as a key priority.^17^ Similarly, 97 people with PPA and 104 care partners in 15 different countries identified ‘having conversations with families and friends’ as the most important when asked what they most wish to change about their lives.^18^ Specifically, people with PPA and their family members in the UK highlighted a need for interventions to help them feel more skilled in conversations.^19^

Communication partner (CP) training (CPT) represents a range of interventions focused on improving the experience of conversations when one person has a communication difficulty.^20^ Independently of individual linguistic or cognitive communication difficulties, CPT draws on retained strengths, such as gesture, to maintain interactional flow.^13 21–23^ It also focuses on interlocutor behaviour. Some family members use effective strategies such as giving time, but others unwittingly expose their partners’ difficulties through the use of ‘barrier’ behaviours. One example is asking of test questions to which the answer is already known,^24^ a pedagogic behaviour used with children but usually avoided in conversations with peers. CPT interventions aim to reduce conversation barriers and increase the use of effective strategies. This enhances conversational skill and confidence and facilitates the flow of natural conversation, thus reducing social isolation.^25^

A recent systematic review of CPT interventions for people with dementia identified 30 studies that suggest this approach could have positive outcomes.^26^ Better Conversations with Primary Progressive Aphasia (BCPPA) was one of the CPT interventions identified in the review. Using the Medical Research Council framework^27^ and informed by the psychosocial model of dementia, behaviour change theory and conversation analysis, the BCPPA CPT intervention was co-developed with people with PPA and their families.^28^ Prior to delivering BCPPA, the speech and language therapist (SLT) video-records a conversation of the person in a natural everyday conversation with their chosen CP (usually a spouse, family member or friend). The SLT then works through four intervention sessions with the dyad (the person and their CP). First, the SLT facilitates a discussion about how conversation works within the dyad, and the dyad is invited to start reflecting on what they think happens in their conversations before viewing 2–3 clips of their conversations. Then, the SLT assists the dyad to analyse these video recordings to identify what they feel might be barriers (behaviours that create problems in conversation such as ‘test questions’) or facilitators (behaviours that help conversation such as using multi-modal communication—speaking, gesture, drawing and other communication aids). The dyad is supported to set goals to identify which behaviours they wish to do more or less of, followed by practising and problem solving these areas. Finally, the SLT supports the dyad to review their goals and consider any future changes in conversations. Importantly, BCPPA is applicable to all major PPA subtypes yet is tailored to the individual needs of the person and their families.

BCPPA was studied as part of a randomised controlled pilot trial across 11 NHS trusts with 18 people with PPA and their family members.^29^ The study showed that SLTs could deliver face-to-face BCPPA in the NHS and that people with PPA and their families found it acceptable. Decisions to deliver BCPPA over four 1-hour sessions was made for pragmatic reasons, but SLT collaborators and participants fed back that some participants would have benefitted from more and others from less. Despite this, results of the study were positive, with outcomes demonstrating participants improved in terms of communication goals, conversation behaviours, confidence and quality-of-life measures. Data from one pair of participants were excluded from the main study because of a subsequent diagnosis of posterior cortical atrophy (PCA) rather than PPA. However, their response to therapy was explored in a single case study. The dyads rated their goals as achieved or overachieved, and the person with PCA improved on confidence, quality-of-life and discourse measures.^30^ However, these two studies also raised questions about when and where this intervention should be delivered (at diagnosis or later; face to face and/or remotely), how many sessions (dosage) are required, how to best measure the effectiveness of the intervention in line with participants’ priorities and whether BCPPA might be acceptable and useful to people with a range of dementia types. Finally, given that in the UK two-thirds of the cost of dementia is borne by family carers, we also had questions about the possible economic implications of BCPPA.

From the outset, this study was informed by guidance from the BCPPA Patient and Public Involvement (PPI) Advisory Group from the prior NHS-embedded pilot study^29^ and designed alongside the BCPPA and other rare dementias PPI Advisory Group convened for this trial. The specific contributions of the PPI advisory groups are outlined in this protocol paper but include providing advice on the funding application, co-designing consent forms and refinement of materials for the programme, as well as providing advice and feedback on all aspects of study management, such as data analysis and dissemination of results.

### Study aims

In line with Medical Research Council guidance on development and management of complex interventions,^27^ this protocol paper describes a phase II mixed-methods randomised controlled pilot and feasibility study with concurrent process and health economic evaluation. The trial will compare the BCPPA CPT intervention to a deferred entry group for people with PPA and other rare dementias and their CPs. In preparation for a future full effectiveness study, the current study will address the following questions:

### Implementation

- What is the optimal schedule and dosage of BCPPA vs treatment as usual?

### Feasibility

- What are the eligibility criteria for the BCPPA intervention?
- Is remote delivery of BCPPA acceptable to people with PPA and other dementias?
- Can the BCPPA be delivered remotely, as intended?
- Do planned outcome measures show sensitivity to change pre-/post-/3 months post BCPPA?
- What are the barriers and facilitators to implementation of BCPPA in an NHS setting?
- What is the optimal sample size for a future effectiveness study?
- What is the most appropriate analysis and method to measure costs and outcomes in a future trial?

## Methods

### Trial design

**Figure 1:**
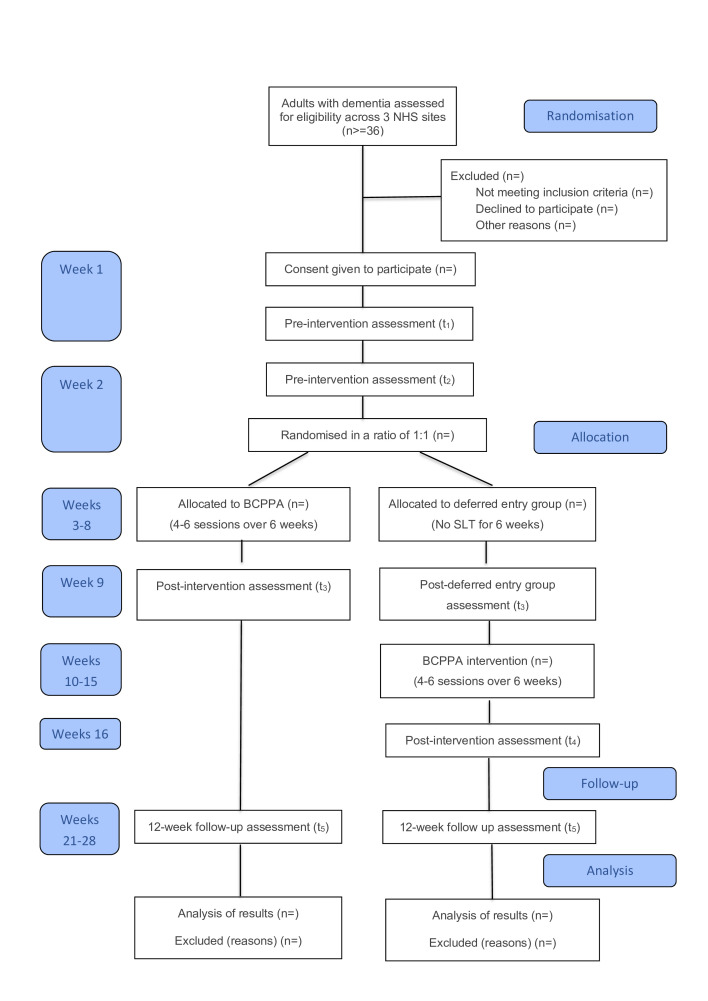
Participant flowchart through BCPPA and other rare dementias study. t_x_, Testing timepoints. BCPPA, Better Conversations with Primary Progressive Aphasia; SLT, speech and language therapist.

This trial was registered retrospectively at ClinicalTrials.gov (ISRCTN16268666). This pilot-feasibility trial employs a randomised controlled design. The protocol follows the Consolidated Standards of Reporting Trials (CONSORT) guidelines, Standard Protocol Items: Recommendations for Interventional Trials (SPIRIT) statement^31^ and the Template for Intervention Description and Replication (TIDieR) checklist and guide.^32^ Comparisons will be made between the treated and the deferred entry groups on pre-treatment versus post-treatment measures. After a 6-week no-treatment phase, deferred participants will then begin intervention. Maintenance will be measured after a 12-week no-treatment period (see figure 1 for an overview of the trial design).

### Participants and recruitment

#### Setting

Participant dyads will be recruited to the pilot feasibility trial from two NHS sites: a dementia diagnostic clinic (which provides services for people living with dementia in urban and rural areas across England) and an outpatient/community speech and language therapist (SLT) service in the east of England. A research assistant, a qualified SLT at the dementia diagnostic clinic or SLTs at the local collaborators will recruit and screen potential participants.

#### Population

All participants with dementia who are referred to the speech and language therapy services described above will be invited to participate in the study based on the inclusion and exclusion criteria to ensure their eligibility (table 1). All healthcare professionals involved in referral and intervention delivery will be invited to participate in semi-structured interviews to explore perceived barriers and facilitators for future implementation. Fortnightly meetings will be held where potential participants will be discussed on a case-by-case basis to determine eligibility. Inclusion and exclusion will be based on clinical review and participant and CP report.

**Table 1:** Inclusion and exclusion criteria

Inclusion criteria	Exclusion criteria
Participants with dementia*	
Have a diagnosis or possible diagnosis of dementia (eg, PPA, FTD, PCA and AD), as identified by a specialist diagnostic dementia service (informed by case history, neurological examination, brain imaging and appropriate psychometric testing if required) and in line with current diagnostic criteria. Have the ability to communicate and understand communication to participate remotely in the BCPPA programme Can see and hear well enough to participate remotely in the BCPPA programme Are functionally able to engage in a remotely delivered BCPPA programme (ie, able to maintain some concentration and remain in a 60–90 min session with support and minimal challenging behaviour that would be unlikely to cause disruption). English as one of their languages of daily use Have a communication partner (CP; family member or friend) who is able to and consents to participating in the project To be confirmed by the RA or local SLT collaborator at the consent appointment: have access to the internet at home with a minimum download speed of 3.8 Mbps and upload speed of 3 Mbps to support 1080 p HD video for group conference calls. (NB: ideally a download speed of 10–25 Mbps and upload speed of 5 Mbps for best results). A home visit or remote internet speed test and advice session by the RA (or local SLT collaborator where appropriate) will be conducted to test and optimise speeds (eg, by troubleshooting, using cabling and cache flushing). Laptops will be loaned to participants who do not have an appropriate PC or laptop available at home.	Do not have capacity to consent Have a prior history of brain lesions or major head trauma Have major physical illness or disability that could affect participation Present with a major psychiatric diagnosis Present with a prominent behavioural disturbance Present with prominent episodic memory, visual memory or visuo-perceptual impairments that would preclude access to telehealth Are unable to demonstrate as a dyad that they can use a computer (PC, laptop or tablet) to access video conferencing (via Zoom) as observed by their performance on a tech screen at the consent session
Communication partners*	
Having identified a potential participant with dementia who fulfils the inclusion criteria outlined above, their communication partners will also be invited to participate in the study in line with the following criteria: Have been identified by the participant with dementia as a communication partner Can attend the appointments and consent to participating in the study Can see and hear well enough to participate remotely in the BCPPA programme Have English as one of their languages of daily use	Have a diagnosis of dementia. As per routine practice, the researchers will not have access to the medical records of the communication partners, and judgements about inclusion and exclusion will be made based on what potential communication partner participants report to the research team. Are unable to demonstrate as a dyad that they can use a computer (PC, laptop or tablet) to access video conferencing (via Zoom) as observed by their performance on a tech screen at the consent session
Healthcare professionals	
Must have been involved in referring people with dementia to the SLT as potential participants in the study, or they are SLT collaborators who delivered the intervention	None

* Eligibility criteria were dependent on the dyad, rather than on the individual.

AD, Alzheimer's disease; BCPPA, Better Conversations with Primary Progressive Aphasia; CP, communication partner; FTD, frontotemporal dementia; HD, high definition; Mbps, megabits per second; PC, personal computer; PCA, posterior cortical atrophy; PPA, primary progressive aphasia; RA, research assistant; SLT, speech and language therapist.

#### Sample size

Based on pilot study findings^29 33^ and NHS site referral rates, we plan to recruit 36 people with dementia and their CPs (usually a spouse, relative or friend), that is, 72 participants in total over a 12-month period. A previous study provided a conservative sample size calculation of 64 people with PPA (and their CPs) based on scores on the Aphasia Impact Questionnaire.^34^ The current pilot will provide information on recruitment and retention to recruit just over half the above estimated sample size in a relatively brief 12-month period. This will facilitate a sample size calculation for a future full trial of BCPPA using the current outcome measures and with participants representing a wider range of dementia types. Recruitment will be reviewed at 2-month intervals during the study, and the recruitment strategy will be amended as necessary to achieve the target number.

### Intervention

The BCPPA intervention^29^ was refined to be delivered online via telehealth in four stages over four to six 1-hour sessions for the purposes of this study (BCPPA and other rare dementias). The prior BCPPA intervention was designed to be delivered face to face over four 1-hour sessions for pragmatic reasons.^28^ Given little is known about the optimal dose of BCPPA, the current study aims to explore the dosage implementation by delivering the intervention over 4–6 sessions. Total number of sessions will be jointly decided by the person with PPA, their CP and the treating SLT, based on their progress in the intervention. People with PPA and their CPs will attend all sessions together. Sessions will be facilitated by the RA or local collaborator SLT via Zoom. Video samples will be made by the participants between the two baseline assessments (to ensure naturalness) prior to intervention. From these recordings, the SLT will assess the dyad’s communication and conversation strengths and needs before commencing the programme. A full description of the BC intervention used in this study is provided in the TiDIER documents (online supplemental appendix).

#### Training of speech and language therapists delivering the intervention

The BCPPA intervention will be delivered by either of the following:

- An RA: a highly specialist, Health and Care Professions Council (HCPC)-registered SLT with experience working with people with aphasia, PPA, rare dementias and AD; the majority (over 80%) of the intervention will be delivered by the RA, or

- A local collaborator (also a specialist, experienced HCPC-registered SLT).

Both the RA and local collaborators will be trained to deliver the intervention by the chief investigator (AV), an experienced SLT and BC trainer. To maximise treatment fidelity and implementation, the chief investigator, RA and local SLT collaborator will have regular documented supervision sessions where video-recorded sessions will be reviewed and any concerns around delivery discussed. They will also have regular contact and ad hoc supervision to ensure knowledge and skills are maintained. Providing close supervision in a pilot trial also aims to overcome local implementation issues, alongside maximising treatment fidelity, and can provide crucial implementation information before moving to a full pragmatic trial.^35^

### Deferred entry group

Participants randomised to the deferred entry group will not receive speech and language therapy for a 6-week period prior to the deferred start of the BCPPA intervention. At present, speech and language therapy services are typically unable to deliver prompt or, in many cases, services for people with dementia who have communication difficulties.^36–38^ Thus, the deferred entry comparator group provides a control condition comparable to a waiting list in an NHS setting.

For the entirety of their participation in the study, both groups will receive all other usual healthcare provision (anticipated to include neurology/psychiatry in dementia diagnostic clinics, general practitioner (GP) reviews and allied health input such as physiotherapy). However, this will exclude any other speech and language therapy interventions until after the 3-month follow-up appointment. At this point, participants will be able to resume all aspects of speech and language therapy provision.

### Randomisation

Each participant will be assigned an anonymised ID on consenting to participate in the study. The principal investigator (AV) will randomise participants using an online random number generator (random.org), at a point when she is masked to case. Randomisation will be stratified by site using blocks of four to ensure a balance in participant randomisation across sites while ensuring balance in overall group sizes. The principal investigator will communicate the outcome of the randomisation to the RA or local SLT following the pre-intervention outcome measures, ensuring that assessors are masked to allocation during the assessment process.

### Allocation concealment and masking

The RA, local collaborators and participants will be masked to treatment allocation during the pre-intervention outcome measures, and outcome assessors administering post-intervention outcome measures will also be masked to allocation. Only single masking will be implemented, as it is not feasible with this and other behavioural interventions to mask the RA or local collaborator delivering the intervention. Participants will be asked not to tell the outcome assessors whether they have had the intervention or not. Outcome assessors will be asked to keep a log of whether they believe they have been unmasked and will receive guidance and training from the RA on how to remain masked.

### Assessment of fidelity

As per gold standard in measurement of treatment fidelity,^39^ all assessment and intervention sessions will be video recorded. At the end of the trial, a random sample of 20% of viable recordings will be analysed using an intervention adherence checklist. Two independent raters will code for adherence to core intervention components and evaluate inter-rater reliability. Consensus methods will be employed where differences are identified. The RA and local collaborators will also be asked to reflect on intervention adherence by completing reflective logs following each session, including documenting session length and tasks completed.

### Assessment of acceptability of the intervention

To inform our understanding of acceptability, anonymised information about diagnosis, time since symptom onset and reason for exclusion will be documented for all potential participants who are not eligible or decline to participate. Acceptability itself will be measured via session attendance and dropout rates, as well as through qualitative feedback from semi-structured interviews. Participating dyads and healthcare professionals delivering the intervention will be invited to participate in semi-structured interviews before and after involvement in the trial. While participants in the current trial will be unable to compare online receipt of the intervention with in-person receipt, a previous pilot study revealed that face-to-face BCPPA was acceptable to participants;^29^ thus, the aim of the current pilot is to determine acceptability of online delivery before proceeding to full trial. The interview topic guide has been informed by the Promoting Action on Research Implementation in Health Services framework^40^ (online supplemental appendix).

### Technical screen and support

A technical screen will be administered during the first baseline session, face-to-face in the participants’ home. This will allow for an assessment of internet functionality and ensure that participants can use Zoom on their own or a loaned device. The technical screen will assess how well dyads can jointly open their device, access a Zoom link and operate the microphone and camera functions. Participant dyads will be asked to navigate the telehealth technology three times with systematic prompts. A measure of responsiveness to prompts will be recorded as time taken and number of prompts required (see online supplemental appendix for the tech screen document). Participants who are unable to navigate the telehealth technology on at least one of the three occasions will be excluded from the study. Additionally, participants will receive information to support access to future remote sessions on Zoom in the form of a co-designed, aphasia-friendly, two-page instruction manual. This was developed in collaboration with the BCPPA and other rare dementias PPI advisory group, detailing how to join and navigate therapy session via Zoom on laptops and iPads. This easy-to-follow manual has also been adapted as a closed caption YouTube video.

### Outcome measures

Outcome measures will be collected at four or five time points depending on randomisation. All participants will complete baseline measures on two occasions prior to randomisation to monitor stability. Participants randomised to the immediate intervention condition will complete measures immediately after the intervention and 3 months after receiving the intervention (ie, four times). After completing the two baseline measure sessions, the deferred group will complete the outcome measures again before, immediately after and 3 months after intervention (ie, five times). Table 2 provides an outline for participants’ schedule of involvement including enrolment, intervention and outcome measurement.

**Table 2:** Schedule of enrolment, interventions and outcome measures

Timepoint	Trial period						
	Enrolment			Post-randomisation			Close-out
	*-t_i_* to 0	*t_1_* *Baseline*	*t_2_* *Baseline*	*t_3_* *Post-treatment/ deferral waiting period*	t_4_* *Deferred entry group post-treatment only*	*t_5_* *12 week post-intervention follow-up*	*t_x_*
Enrolment							
Eligibility screen	X						
Informed consent		X					
Baseline assessments		X	X				
Randomisation			X				
Intervention/comparator							
*BCPPA*			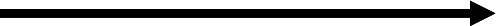				
*Deferred entry group*				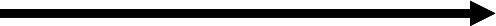			
Assessments							
Primary outcome (*CCRSA*)		X	X	X	X*	X	
Primary outcome (*CPIB*)		X	X	X	X*	X	
Secondary outcome (*CAT subtests*)				X	X*		
Secondary outcome (c*onversation samples*)		X	X	X	X*	X	
Secondary outcome (*GAS*)		X	X	X†	X†	X	
Secondary outcome (*personal narrative*)		X	X	X	X*	X	
Secondary outcome (*picture description*)		X	X	X	X*	X	
Secondary outcome (*PACT*)		X	X	X	X*	X	
Secondary outcome (*QCPR*)		X	X	X	X*	X	
Health economics (*EQ5-5D-5L+C*)		X	X	X	X*	X	
Health economics (*ICECAP-O*)		X	X	X	X*	X	
Health economics (*RUD*)		X		X			
Semi-structured interviews			X				X

* Deferred entry group assessment only.

† Completed as part of the intervention in both conditions (ie, immediate treatment and deferred entry group).

BCPPA, Better Conversations with Primary Progressive Aphasia; CAT, Comprehensive Aphasia Test; CCRSA, Communication Confidence Rating Scale for Aphasia; CPIB, Communicative Participation Item Bank; EQ5-5D-5L+C, EuroQol 5-Dimension 5-Level questionnaire plus Cognition; GAS, Goal Attainment Scaling; ICECAP-O, Investigating Choice Experiments for the Preferences of Older People; PACT, Progressive Aphasia Communication Tool; QCPR, Quality of the Carer-Patient Relationship Scale; RUD, Resource Utilisation in Dementia; t_x_, testing timepoints.

The combined time for completing the measures should not exceed 90 min, including greetings, rapport building, technical set-up and switching between tests. Pre-intervention measures will be administered by the RA or a local SLT collaborator, while all post-intervention or deferred entry period and 3-month follow-up measures will be administered by an outcome assessor (an SLT student with training in the assessment of people with PPA and dementia) but crucially masked to treatment group allocation (immediate or deferred entry group).

During the BCPPA intervention, participant dyads will also complete Goal Attainment Scaling^41^ to determine specific individualised targets for the intervention and measure change in these targets. With support from the SLT, participant dyads will identify goals and weigh the goals according to their importance and likelihood of being achieved. At the end of therapy, the goals will be reviewed by the participant dyads with the support of an SLT and an outcome score agreed.

Outcome measurement tools described in table 3 were selected based on a recent international core outcome set for PPA.^18^ To capture the top construct from the core outcome set for PPA (*participate in conversations with family and friends)*, two primary outcome measures were selected: the Communication Confidence Rating Scale for Aphasia (CCRSA)^42^ and the Communication Participation Item Bank (CPIB).^43^ The CCRSA captures participants’ confidence in communication activities including conversations, whereas the CPIB captures participants’ ratings of their ability to participate in conversations. The CCRSA has been shown to demonstrate change in confidence over time in previous PPA intervention studies (eg, ^44–46^) including the prior BCPPA pilot study.^29^ However, the CCRSA has only been validated for people with stroke aphasia,^42^ and there are no published psychometric studies on its use in PPA. The CPIB has also been used in previous PPA intervention studies.^44 45^

**Table 3:** List of outcome measures and their alignment with the core outcome set for PPA constructs^18^

Measure	Description	Approximate administration time and to whom	Alignment with the COS PPA constructs
Primary outcome measures			
Communication Confidence Rating Scale for Aphasia (CCRSA)^42^	A 10-item questionnaire whereby the participant rates their confidence on a Likert scale from 0 to 100	15 min; person with dementia only	Participate in conversations with family and friends
Communication Participation Item Bank (CPIB)^43^	Communication Participation Item Bank (CPIB)^43^	10 min; person with dementia only	Participate in conversations with family and friends
Secondary outcome measures			
Comprehensive Aphasia Test (CAT)^47^	Three language subtests comprising spoken sentence comprehension, spoken object naming and picture description subtests	15 min; person with dementia only	Get words out, be more fluent and understand what others are saying
Personal narrative	Talking on a topic of interest elicited with *“*Tell me about something that is personally meaningful to you*”* with examples and prompts	5 min; person with dementia only	Get words out and be more fluent
Conversation samples	In the first assessment session, participants will be recorded having a 10 min (approximately) conversation. Participants will then make three 10 min video recordings of natural conversation using a loaned iPad and stand before the second assessment. This will be repeated at all other assessment timepoints.	10 min; both the person with dementia and their communication partners	Participate in conversations with family and friends and convey a message by any means
Progressive Aphasia Communication Toolkit (PACT)^52^	PACT ratings are done by an independent assessor, masked to timepoint, based on video-recorded discourse and conversation samples.	No additional minutes. PACT ratings are done by an independent assessor, masked to timepoint, based on video-recorded discourse and conversation samples of the person with dementia.	Get words out, be more fluent, convey a message by any means and understand what others are saying.
Goal Attainment Scaling (GAS)^41^	GAS is completed during the intervention itself and reviewed at the end to determine specific individualised targets for the intervention and measure change in these targets.	No additional minutes; both person with dementia and their communication partners	
Quality of caregiver-patient relationship (QCPR)^53^	A questionnaire designed to evaluate relationship and completed by both the person with dementia and their carer. The rating scale requires participants to rate 14 statements on a 1–5 scale from totally agree to totally disagree.	10 min; both person with dementia and their communication partners	

COS PPA, core outcome set for primary progressive aphasia.

Additional secondary outcome measures were selected to understand the impact of the intervention on language and discourse skills. These were the spoken-sentence comprehension, spoken-object naming and picture-description subtests from the Comprehensive Aphasia Test (CAT).^47^ A personal narrative was identified as the most valid elicitation task for generating spoken-discourse samples in best practice guidelines for reporting spoken discourse in neurogenic communication disorders.^48^ It is also identified as one of the most ecological and functional methods of evaluating discourse in a systematic review of discourse measures.^49^ A personal narrative also provides a more naturalistic, less constrained discourse sample to contrast with the highly structured and constrained sample collected via the CAT picture-description task.

Conversation samples were selected as further secondary outcomes measures both to enable the measurement of change in conversation behaviours (both in the person with dementia and their CPs) and as the most proximal measure to the BCPPA intervention. Video recordings of natural conversations between the person with dementia and their CPs will be coded by raters masked to pre-intervention/post-intervention timepoints. They will identify and count conversation behaviours using an approach developed for use in BC intervention studies^50^ and previously used in the BCPPA study.^29^ Given the time-consuming nature of this outcome measure, a comparable clinical measure, the Kagan Measure of Skill in Conversation–Dementia and Measure of Participation in Conversation–Dementia Scale,^51^ will also be used for comparison. Video recordings will also be used for masked assessors to administer the Progressive Aphasia Communication Toolkit (PACT).^52^ PACT is designed for people living with conditions that result in progressive communication change, such as dementia and PPA. PACT documents and monitors symptom progression or treatment responsiveness using qualitative report and scales that evaluate strengths and needs across the communication domains: speech-voice, language, social-pragmatics and discourse.

Quality of caregiver–patient relationship^53^ measures change in both participants’ relationship to one another. Relationship was identified by the PPI advisory group as an important domain to consider.

### Process evaluation

Following the intervention, participants with dementia and their CPs will be asked to participate in semi-structured interviews, structured using the Normalisation Process Theory,^54 55^ to capture perceived barriers and facilitators for future implementation (see topic guide in online supplemental appendix). All healthcare professionals involved in referring people with dementia to the study or delivering the intervention themselves (local SLT collaborators) will also be asked to participate in the interviews, also structured using the Normalisation Process Theory, to capture perceived barriers and facilitators for future implementation (see draft topic guide in online supplemental appendix). These interviews will be conducted after they have delivered the intervention and will be conducted on Zoom, lasting for up to 90 min.

### Health economic evaluation

The health economic analysis will determine the most appropriate perspective of analysis and methods of measuring and valuing costs and outcomes in a future cost-effectiveness analysis of BCPPA versus usual care. The Health Economics Analysis Plan (HEAP) will set outline objectives and methods for data collection and analysis, and data will not be shared with the health economics researchers until the HEAP has been reviewed and approved by the lead health economist (co-author RTE). To explore the feasibility of adopting both a health and social care perspective and wider societal perspective, we will collect data using the EuroQol 5-Dimension 5-Level^56^ to derive quality-adjusted life years, the Investigating Choice Experiments for the Preferences of Older People ^57^ to capture wider capability-based quality of life, and a validated questionnaire, the Resource Utilisation in Dementia^58^ to capture service utilisation costs (see table 4 for a summary of health economic measures). The economic analysis will explore the completeness and responsiveness of the health economic measures to inform the design of a future definitive cost-effectiveness analysis.

**Table 4:** List of health economic measures

Health economics measures		
Measure	Description	Approximate administration time
EQ5-5D-5L+C^56^ A brief health-related quality-of-life measure with cognition.	A 6-item questionnaire designed to evaluate health-related quality of life. This will be completed by both the person with dementia and their partners.	5 min/person
Investigating Choice Experiments for the Preferences of Older People - CAPability index (ICECAP-O)^57^ Has been identified as the health economic measure that most closely aligns with the constructs identified on the core outcome set for PPA.^18^ Has been used in previous speech and language conversation intervention studies^66^	A 10-item questionnaire designed to evaluate capability. This will be completed by both the person with dementia and their partners.	5 min/person
Resource utilisation in dementia (RUD)^58^ questionnaire The RUD is a validated instrument for assessing resource use (community and hospital-based resource use).	A questionnaire asking the participant to outline the number of healthcare contacts and service use (eg, admissions/GP appointments) over a set time period. To be completed by the carer only, this measure captures service use of both the person with dementia and their carer.	15 min

GP, general practitioner.

### Data collection and management

Personal information (including date of birth, sex, diagnosis, symptom onset and diagnosis, occupational and educational background, as well as relationship between participant dyads) will be collected from consenting participants and will remain confidential. Participants will be given a unique number that will be used on all paperwork, assessment score sheets, video and data files and all subsequent analysis documents and publications. Lists of participant names and their unique numbers (required to conduct the remote randomisation procedure) will be kept by the chief investigator at University College London (UCL) and the RA or designated local collaborator in a locked cabinet at each NHS site. Each list will only contain the names of participants based at the relevant site.

Participants will consent to be video recorded during conversations for the purposes of outcome measurement and fidelity rating and to provide clips for video feedback during intervention sessions. Semi-structured interviews will also be video recorded. All transcripts of conversation data will be anonymised using pseudonyms for named people and places. One aspect of this research study involves the detailed analysis of video recordings where faces will be fully visible. It is not appropriate to blank out faces as people’s expressions form a significant part of human communication: the focus of the intervention being piloted. If participants consent to use of recordings at conferences or in teaching, their faces will be blurred using video software. Additionally, participants (people with dementia and their CPs) will be contacted at the end of the study to re-visit consent for use of specific video clips for this purpose. No semi-structured interviews with participants or healthcare professionals will be used for the purposes of teaching or at conferences. Only the research team members will have access to the entire video-recorded dataset.

It is common practice for SLTs to ask patients to video themselves. Many patients now have their devices and will often do this. We have followed this common practice. Transfer of patient data from NHS sites to the university will be managed electronically via the secure Data Safe Haven system where site technology supports this. Where this is not possible, the chief investigator or RA will collect data from outcome assessors and local collaborators and upload it to the secure Data Safe Haven system.

The study is compliant with General Data Protection Regulations. If information disclosed by any participant leads the first author to believe that any person is at risk of harm, confidentiality will be breached to ensure safety. Given this is an NHS-embedded pilot study, delivered by local SLT collaborators, no additional data monitoring committee has been established beyond the sponsor and the BCPPA steering group. The BCPPA steering group is comprised of a PPI representative, two senior SLTs and a clinical psychologist, a professor of neurology and an associate professor in speech and language therapy. This group is independent from the sponsor and funder and meets four times annually to discuss recruitment, retention and data management.

All electronically held data including video recordings will be stored for 10 years on the UCL Data Safe Haven. This is in accordance with current University College London Records Management Policy; research findings will need to be stored by UCL as sponsor for 10 years after the research has finished. Depending on which consent option has been chosen by the participants with dementia or their consultee, and their CPs, all video data collected during the trial will also be archived on the CAVA repository. This procedure is standard practice in the Department of Language & Cognition, UCL, for video data collected from people with communication disorders including aphasia, dementia and Parkinson’s for research purposes, and has been approved by NHS ethics committees in the past for research led by the chief investigator.

### Analysis plan

Qualitative and quantitative analysis will be used for this single masked, randomised controlled pilot study. Descriptive statistics will be used to report recruitment, attendance, attrition and reasons for dropout. A CONSORT^59^ and SPIRIT ^60^ flow chart will be used to present overall recruitment to and progression through the study (figure 1). Recorded intervention sessions will be transcribed, and descriptive statistics will be used to analyse dosage. Data collated from potential and actual participants, including date of onset, will also be examined to explore patterns related to optimal dosage and schedule of the intervention. Recruitment and retention rates will be used to support sample size calculations to inform a future full trial and plan the required number of sites to meet this target. Adverse events will also be recorded, and participant interviews examined to inform future recruitment procedures.

All outcome data will be explored to test for assumptions of normalcy. If assumptions are not met, appropriate non-parametric tests will be used. Repeated-measures analyses of variance will be used with group (immediate vs deferred) as a between-subject factor and time (pre-therapy and post-therapy) as a within-subject factor. Results will be used to determine a suitable and sensitive outcome measure and perform a power calculation to determine the sample size for a fully powered trial. Conversation data will be analysed following a procedure developed for the BCA intervention^29^ to identify change in the use of targeted strategies following intervention. This will involve counting barrier and facilitator behaviours in 5 min video samples selected from the pre-intervention and post-intervention conversation samples. These counts will be analysed using Poisson trend tests suitable for observations occurring in Poisson distribution. All pre-therapy conversations will be weighted the same as one another, as will all the post-therapy conversations. Outcomes for different participants will be investigated using a test for homogeneity, and where this is significant, the effect for different dyads will be calculated using the Holm–Bonferroni procedure.

Analysis of fidelity and adherence data will inform ongoing training needs. Although fidelity data are sparse for speech and language intervention trials, these processes, deployed in our previous BCPPA study, achieved 87.2% fidelity.^39^ Thus, we have selected 80% as the minimum target for fidelity.

Semi-structured interviews will be recorded and transcribed, and reflexive thematic analysis^61^ will be used to examine acceptability of the intervention across participants living with PPA or other rare dementias. Semi-structured interviews with healthcare professionals and collaborators involved in the recruitment of participants (referral to an SLT) and intervention delivery will be recorded and transcribed. Codebook analysis, informed by implementation frameworks, will be conducted to explore the barriers and facilitators to accessing and delivering the intervention.

### Criteria for success

This research will be considered appropriate to proceed to a full effectiveness study if:

- The RA, local SLT collaborators and participants report positive views on remote delivery of BCPPA and other rare dementias.
- Participant recruitment and retention targets are achieved within the designated time frame.
- An appropriate outcome measure is determined and sample size estimated.
- An appropriate perspective of analysis and measurement of costs is determined for a future cost-effectiveness analysis.

### Patient and public involvement

This study is born out of PPI work undertaken with people with PPA and their families during the first BCPPA pilot study^29^ and recent work with a wider representative group of people affected by dementia. Importantly, all stakeholders felt strongly that this research was important to improve communication, mental health and relationships for people with PPA and their families. They felt that this intervention has the potential to improve quality of life and keep people living at home for longer. In collaboration with the current members of the BCPPA and other rare dementias PPI advisory group, participant information forms and consent forms were co-produced for this study. Decision-making on aspects of the ethic application was informed by PPI work (eg, discussion of risk management and consent pathways) as per guidance being developed on the Trial Methodology Research Partnership complex consent pathways working group (of which the chief investigator (AV) is a member). All topic guides for interviews were co-produced, and outcome measures for the trial were identified from a participant-led core outcome set for PPA project undertaken by the chief investigator,^18^ as recommended by the previous BCPPA PPA advisory group as essential to inform the choice of measures for the current trial.

The BCPPA and other rare dementias CPT intervention was jointly refined with members of the BCPPA and other rare dementias PPI advisory group to ensure it can be delivered remotely and is applicable to the wider participant group. This was undertaken over three co-planned full day co-design meetings at UCL. All participants were invited to participate in BC training in the university student-led clinic prior to the meetings, ensuring they had relevant experience of the intervention. Consensus methods such as nominal group technique style voting approaches (known to enable people with communication difficulties to participate on equal terms) and supported communication (pictures, writing, individual and group discussion) were essential approaches for the discussions around refinement. No changes were made in the delivery of the core components, rather the changes focused on two main areas:

1. Preparing and supporting participants to attend therapy online via Zoom: while provision was made to loan laptops to participants who do not have appropriate devices available, the PPI advisory group advocated for training and support for participants to access the telehealth platform (Zoom). PPI advisory group members worked with the first author (RT) and two junior researchers (LR and RK, student SLTs) to inform the development of a prototype screening tool to assess the technology skills of the participants and instructional guidance sheet on video recording and using telehealth during the study. The PPI advisory group then provided feedback for further refinement of the screening tool and instructional guidance.
2. Changing the technical terminology to ensure accessibility throughout: specifically, the terminology used to refer to conversation and communication difficulties was refined to make provision for people with non-PPA dementias. Additional recommendations were also integrated such as using *tips* and *hints* instead of strategies and *rewarding activities* instead of homework practice.

Future plans for ongoing involvement of the BCPPA PPI advisory group include co-analysis of anonymised data collected in the study and planning and delivering dissemination activities such as presenting at academic conferences, PPA support group meetings, podcasts and co-writing peer-reviewed journal articles.

## Ethics and dissemination

This study will be conducted in line with the Declaration of Helsinki and ethical approval was granted by the Health Research Authority for England and Wales IRAS Project ID: 341322 REC REF 24-NI-0123. A minor amendment to add the PACT outcome measure was approved on 17 October 2025 (ref EA-102907). This study is sponsored by the University College London Joint Research Office, UCL, Gower Street, WC1E 6BT. Results from this study will be shared at conference, published in peer-reviewed journal articles and shared with participants and PPI advisors in accessible coproduced formats.

### Informed consent

All practicable support will be provided to assist potential participants, including appropriate care and facilitation to explain the research and support understanding, and sufficient time to allow prospective participants to reach a decision. Participants will be excluded if they lack capacity to consent at the outset of the study. The BCPPA and other rare dementias intervention is relatively brief (3–6 weeks), and the total period of involvement in the study is maximally 31 weeks. It is not anticipated that participants’ decision-making capacity will change significantly during this period. However, this will be reviewed at every contact over the 31-week duration of their involvement. As per current Medical Research Council and National Institue for Health and Care Research (NIHR) Complex and alternative consent pathway guidance, no participant will be excluded from the study just because they no longer have capacity, and where participants’ decision-making capacity changes, their CPs will be invited to give their opinion as a consultee at this time.

Informed consent will be obtained by the RA at the primary research site or by the local collaborator (SLT) delivering intervention at the participating sites where therapy is being delivered. The current guidance from the Mental Capacity Act 2005^62^ and Royal College of Speech and Language Therapists^63^ regarding gaining consent from people with communication difficulties will be followed. The chief investigator, RA and local SLT collaborators all have considerable experience of working with individuals with communication and cognitive impairment. They also complete annual mandatory training on the Mental Capacity Act 2005^62^ and issues related to obtaining consent. All team members, including junior researchers involved in the study, must participate in a training session about consent processes. This will include problem-based support on a case-by-case basis throughout the study. If any team members have any doubts regarding the capacity of a person with dementia to provide informed consent, advice will be sought from the chief investigator, RA, or as per standard practice, an appropriate clinician who is familiar with the participant (eg, GP).

Participant information sheets and consent forms have been designed to be dementia and aphasia friendly to support the process of gaining informed consent. These have been designed using a resource for researchers in communication disability ‘engaging people who have aphasia’^64^ and modified with advice from people with dementia and their carers in the PPI advisory group. Throughout the interactions with all participants, clinicians will speak slowly and clearly; repeat information where necessary and ask open as opposed to closed questions.^65^ Importantly, as per the opinions of the PPI advisory group, people with dementia and their CPs will consent to accessible forms to ensure equity in the consent process.

It will be made clear to participants that no disadvantage will accrue if they choose not to participate. Consent has to be regarded as a continuing process rather than a one-off decision, and willingness to continue to participate in the study will be continually checked through discussion with participants. Should any person with dementia or their CPs show verbal or non-verbal indications of refusal or reluctance to participate in sessions or assessments, the CI, RA or local collaborator will revisit their decision to be involved in the study. Should they continue to decline participation in the study, then they will be withdrawn.

In line with the Mental Capacity Act 2005,^62^ MCA Code of Practice 2007^65^ and advice from the project PPI advisory group, the process for obtaining consent for each potential participant, both people with dementia and their CPs, will be as follows:

1. The potential participants (participants with dementia, their CPs and healthcare professionals) will receive the ‘Participant Information Sheet’ about the study from the recruiting RA or local SLT collaborator by email or equivalent (eg, posted or handed to them) at least 48 h prior to a face-to-face discussion with them.
2. The participants and RA or local SLT collaborator will meet face-to-face to discuss the Participant Information Sheet and explain the project in more detail, giving the opportunity for the person to ask questions.
3. The RA or local SLT collaborator will judge the quality of the person’s decision to participate in the study by asking the person to tell them about the purpose of the study, reason they have arrived at their decision and reasons for and against participating in the study. If the person is able to reach a decision about agreeing or refusing to participate in the study, the clinician will accept the person’s decision. They will make it clear that the person has the right to refuse to participate or withdraw from the study at any point and that this will not disadvantage them in any way. Participants will be reminded about their right to withdraw by the chief investigator, RA or local collaborator during every contact point throughout the study.
4. If the person decides to participate in the research and they are judged as having the capacity to make this decision, they will be asked to sign and return the participant consent form to the RA. Each participant will be given a copy of the information sheet and sign the consent form for keeping. The original copy of the consent form will then be kept in their paper file in a locked cabinet at UCL. A digital copy of the consent form will also be made and stored on an encrypted external hard drive for study purposes.

In line with the MCA 2005, and in the unlikely event that a participant’s level of impairment increases significantly during the period they are involved in the study, and changes in capacity to consent to participation were to occur, the following process will be followed:

1. If the RA, local SLT collaborator or junior researcher has any doubts regarding the capacity of a person with dementia to provide informed consent, capacity to consent will be revisited by the RA or local SLT collaborator. As per current MRC and NIHR Complex and alternative consent pathway guidance, no participant will be excluded from the study just because they no longer have capacity, and where participants’ decision-making capacity changes, their CPs will be invited to give their opinion as a consultee at this time. In line with the MCA 2005,^62^ the consultee by virtue of having been recruited as a CP is defined as ‘interested in P’s welfare’.
2. CPs who are prepared to be consulted will be invited to consider what they think the views of the person with dementia would have been if they had capacity. They will be given a 'consultee’ information sheet and asked to complete the consultee declaration for the continued participation of their relative in the research.
3. The CP (consultee) will be able to withdraw the person with dementia from the study at this time or any time after this.
4. Any person with dementia showing verbal or non-verbal indications of refusal or reluctance to participate in the focus group will be withdrawn from the study.

Should any person with dementia or their CPs show verbal or non-verbal indications of refusal or reluctance to participate in sessions or assessments, the chief investigator, RA or local SLT collaborator will revisit the participant’s decision to be involved in the study. Should they continue to decline participation in the study, then they will be withdrawn. If the partner of a person with dementia withdraws from the feasibility trial, then the person with dementia will have to withdraw also, as the intervention is completed together.

The RA or local collaborator will explain that participants are under no obligation to enter the trial and that they can withdraw at any time during the trial, without having to give a reason and without prejudicing his/her further treatment. Data and samples collected up to the point of withdrawal can only be used after withdrawal if the participant has consented for this. If a participant is required to re-consent or new information is required to be provided to a participant, the chief investigator must ensure that this is done in a timely manner.

## Discussion

This phase II mixed-methods process evaluation study in the form of a randomised controlled feasibility study comparing the BCPPA CPT intervention to a deferred entry group for people with PPA and other dementias and their CPs will inform a future full effectiveness study. Although a recent systematic review of CPT for dementia identified five randomised controlled trials,^26^ none of these described CPT interventions were delivered to both the person with dementia and their CPs; rather, these interventions focused on training solely the care partner. Other than the BCPPA pilot study^29^ that preceded this study, to our knowledge, no previous randomised controlled trials have explored the effectiveness of CPT for people with dementia.

The previous BCPPA pilot study demonstrated that a sample size calculation for a future effectiveness study would need to recruit at least 64 participants to demonstrate a reduction in the impact of aphasia on the measurement tools used in that study (using the Aphasia Impact questionnaire^34^). Importantly, participants and SLTs delivering the intervention reported that this measure did not accurately represent the full benefits of the intervention. This study is therefore informed by recent core outcome sets for PPA that highlighted the importance of ‘having conversations with families and friends’ as a priority outcome.^18^ This study will trial a range of outcome measures that aim to capture these constructs. We anticipate that the inclusion of a double baseline and 3-month post-intervention follow-up will provide a more accurate representation to inform the selection of a primary outcome measure and a future sample size calculation. Unfortunately, only one of the measures being employed in this study has been designed for and with people with PPA and other rare dementias. To counter this, several measures have been selected, and amendments were made to include the most up-to-date measures where possible.

The results of this study will be disseminated via presentations at national and international conferences and submitted for publication in peer-reviewed scientific journals. With support from the study steering committee, results will be disseminated through professional and user group networks via publications and presentations, for example, at the PPA support group branch of the Rare Dementias Support Group based at UCL (http://www.raredementiasupport.org/) and via the annual international PPA awareness campaigns (https://speechtherapyppa.com/ppa-awareness-day).

Given the advent of disease-modifying interventions for AD that at present have the potential to support people to live between 6 and 18 months longer, there is an urgent need for interventions that can also support people to live well. We anticipate that future studies might need to explore the combined impact of disease-modifying pharmaceutical options alongside non-pharmacological psychosocial treatments such as CPT to provide optimal outcomes and genuinely reduce the risk of negative health outcomes.

## Supplementary material

10.1136/bmjopen-2026-117509 online supplemental file 1

10.1136/bmjopen-2026-117509 online supplemental file 2

10.1136/bmjopen-2026-117509 online supplemental file 3
