# Supplementary material for: ‘Better Conversations with Primary Progressive Aphasia and other rare dementias (BCPPA plus)’ telehealth communication partner training programme: protocol for an NHS embedded randomised controlled feasibility and implementation study
Source: BMJ Open. 2026 Aug 7;16(8):e117509. doi: 10.1136/bmjopen-2026-117509 (PMC13475093; doi:10.1136/bmjopen-2026-117509)
Supplement: Supplementary file 1 [file bmjopen-16-8-s003.pdf]

Semi-structure interviews (Implementation)
(Based in the PARIHS framework, Bergström et al, 2020)

Topic Guides

For speech and language therapists:

1. What do you know about the speech and language therapy trial of the BCPPA intervention. Do you think it is a rigorous and robust intervention that makes sense?
2. In your opinion, do you think the intervention will work for the people who receive it?
3. Does the intervention fit with your local setting/service?
4. Do you think the intervention is accessible and usable?
5. Do you think the intervention requires significant changes in how you deliver care?
6. What are the implications in terms of your scope of service or scope of practice?
7. Have you had to learn anything new to deliver it?
8. Does it have advantages over what you were doing before?
9. Do you think it will enhance the patient experience?
10. Do you think it will reduce barriers to day-to-day care for the patients and their families?
11. Do you feel the service you work in want new speech and language therapy interventions for people with dementia?
12. Are there differences of opinion about the utility of speech and language therapy in the team you work in?
13. Do you think you will be able to deliver the therapy in practice?
14. Will you use the therapy going forward?
15. Will the team need more training going forward?
16. Does your team have the funding, time, support and permission to keep using this therapy?
17. Do you think speech and language therapy interventions such as this will be supported by services/managers/therapists in the future?
18. Do you have past experiences of implementing changes that influence how you feel now?
19. Are there mechanisms in place to help you keep using this therapy?

For multidisciplinary team:

1. Do you feel there is a need for new speech and language therapy interventions for people with dementia?
2. What do you know about the speech and language therapy trial of the BCPPA intervention. Do you think it is a rigorous and robust intervention that makes sense?
3. Does the intervention fit with your local setting/service?
4. Are there differences of opinion about the utility of speech and language therapy in your profession or your service or both?
5. Do you think the intervention requires significant changes in how you refer or use speech and language therapy?
6. In your opinion, do you think the intervention will work for the people who receive it?
7. Do you think it will enhance the patient experience?
8. Do you think it will reduce barriers to day-to-day care for the patients and their families?

9. Do you think speech and language therapy interventions such as this will be supported by services/managers/therapists in the future?

Questions for the people with dementia and their families:

Tell me about the following:

1. Would you like your conversations together to change?
2. Do you need your conversations together to change?
3. What makes you want to do this therapy?
4. Do you think it will be / was worth doing this therapy?
5. Do you think you will be able to do the therapy? Were you able to do the therapy?
6. Do you think you'll be able to make a long-term change in your communication and conversations?
7. Do you both feel you have learnt enough from therapy to make changes to your communication?
8. Do you think you will need more therapy and what will you need going forward?

Ref:

Bergström, A., Ehrenberg, A., Eldh, A. C., Graham, I. D., Gustafsson, K., Harvey, G., ... & Wallin, L. (2020). The use of the PARIHS framework in implementation research and practice—a citation analysis of the literature. *Implementation Science*, *15*, 1-51.
