# Supplementary material for: ‘Better Conversations with Primary Progressive Aphasia and other rare dementias (BCPPA plus)’ telehealth communication partner training programme: protocol for an NHS embedded randomised controlled feasibility and implementation study
Source: BMJ Open. 2026 Aug 7;16(8):e117509. doi: 10.1136/bmjopen-2026-117509 (PMC13475093; doi:10.1136/bmjopen-2026-117509)
Supplement: Supplementary file 2 [file bmjopen-2026-117509.reviewer_comments.pdf]

PEER REVIEW HISTORY

BMJ Open publishes all reviews undertaken for accepted manuscripts. Reviewers are asked to complete a checklist review form and are provided with free text boxes to elaborate on their assessment. These free text comments are reproduced below.

ARTICLE DETAILS

Title (Provisional)

The 'Better Conversations with Primary Progressive Aphasia and other rare dementias (BCPPA plus)' telehealth communication partner training program: Protocol for an NHS embedded randomised controlled feasibility and implementation study

Authors

Talbot, Richard; Keogh, Roisin; Rosenthal, Lola; Anthony, Bethany; Pearson, Mark; Edwards, Rhiannon Tudor; Henry, Maya L.; Warren, Jason; Varley, Rosemary; Advisory Group, BCPPA Patient and Public Involvement; Volkmer, Anna

---

VERSION 1 - REVIEW

**Reviewer**	**1**
**Name**	**Buskens, Erik**
**Affiliation**	**University of Groningen, University Medical Center Groningen, Epidemiology**
**Date**	**04-May-2026**
**COI**	**None**

The topic is highly relevant, particularly given the scarcity of RCTs for such interventions in dementia. The trial design is generally well described, and the selected outcomes are generally appropriate.

Of note: this technically is not a cross-over study. One cannot go back to not having undergone the intervention.

This intervention by definition will be delivered 'online', so how does this relate to the 3rd objective/RQ?

The 4th RQ 'What is the best way to measure...' is vague. Please rephrase and or elaborate.

It would appear that more than the initially put forward RQs are being studied. These only appear upon reading the entire manuscript. Since this iis a pilot study this may be okay but please do elaborate from the beginning. Eg. ' All health care professionals involved in referral and intervention delivery will also be invited to participate in semi structured interviews to

explore perceived barriers and facilitators for future mplementation. Fortnightly meetings will be held where potential participants will be

discussed on a case-by-case basis to determine eligibility.' Also 'As per gold standard in measurement of treatment fidelity...' and 'Quality of caregiver patient relationship (Spruytte et al, 2002) measures change in both participants' relationship to one another.'

Of a more technical note:

Heterogeneity of the Patient Population

The inclusion criteria appear broad, covering different dementia types (e.g., PPA, FTD, AD), distinct symptom profiles and progression patterns. Further justification for combining these populations is needed.

It would also be important to clarify whether disease severity will be recorded (e.g., cognitive impairment level, CDR score), and whether this will be accounted for in the analysis.

Sample Size and analysis plan

The proposed sample size (n=36) appears small relative to the breadth of the research questions, particularly given the plan of quantitative approach.. The study aims to address multiple dimensions (implementation, feasibility, and future cost-effectiveness considerations), which will likely require subgroup analyses.

Given the heterogeneity in both patient population (e.g., dementia subtype) and intervention delivery (e.g., dosage and schedule of BCPPA), further stratification may substantially reduce statistical power and increase the risk of selection bias. The use of repeated measures ANOVA (with group as a between-subject factor and time as a within-subject factor) may not be robust in the context of small sample sizes and potential missing data. Prior literature suggests that substantially larger samples (~114 participants) may be required for adequate statistical power in similar designs (Osstyn, S. L., 2026).

If feasible, the authors may consider increasing the sample size, or narrowing this particular study's aims (e.g., focusing more explicitly on feasibility or incorporating a stronger qualitative component)

Outcome Measures

While the selected outcomes are appropriate, the progressive nature of dementia raises questions about the expected duration of effects. The authors may consider whether long-term outcomes will be expected and if that should be assessed.

In addition, given the dyadic nature of the intervention and the stated aim to explore perspectives relevant to future economic evaluation, the inclusion of caregiver-reported outcomes (e.g., subjective burden, quality of life) should be considered.

Osstyn, S. L., Handels, R., Rupprecht, C., Boots, L. M. M., & de Vugt, M. E. (2026). Cost-consequence analysis of the partner in balance program for informal carers of persons with dementia. Aging & Mental Health, 30(3), 553–562. https://doi.org/10.1080/13607863.2025.2573845

VERSION 1 - AUTHOR RESPONSE

**Reviewer: 1 Dr. Erik Buskens, University of Groningen, University Medical Center Groningen**	
1. The topic is highly relevant, particularly given the scarcity of RCTs for such interventions in dementia. The trial design is generally well described, and the selected outcomes are generally appropriate.<br>Of note: this technically is not a cross-over study. One cannot go back to not having undergone the intervention.	Many thanks. The study design has been amended to the appropriate terminology, and the control group has been described as the deferred entry group.
2. This intervention by definition will be delivered 'online', so how does this relate to the 3rd objective/RQ?	Whilst participants in the current trial are unable to compare online receipt of the intervention to in person receipt, the previous pilot study evidenced that face to face BCPPA was acceptable to participants (Volkmer et al, 2023), thus the aim of the current pilot is to determine acceptability of online delivery before proceeding to full trial. We have added this statement to the methodology section under the “assessment of acceptability of the intervention” title. The abstract and introduction have also been amended to make it clear that the previous pilot was face to face.
3. The 4th RQ 'What is the best way to measure...' is vague. Please rephrase and or elaborate.	RQ 4 has been rephrased to make it less vague:<br><br>“Do planned outcome measures show sensitivity to change pre-/post-/3-months post BCPPA?”
4. It would appear that more than the initially put forward RQs are being studied. These only appear upon reading the entire manuscript. Since this iis a pilot study this may be okay but please do elaborate from the beginning. Eg. ' All health care professionals involved in referral and intervention delivery will also be invited to participate in semi structured interviews to explore perceived barriers and facilitators for future implementation. Fortnightly meetings will be held where potential participants will be discussed on a case-by-case basis to determine eligibility.' Also 'As per gold standard in measurement of treatment	The RQs have been refined to reflect the reviewers suggestions:<br><br>What are the eligibility criteria for the BCPPA intervention?<br><br>Is remote delivery of BCPPA acceptable to people with PPA and other dementias?<br><br>Can the BCPPA be delivered remotely, as intended?

	fidelity...' and 'Quality of caregiver patient relationship (Spruytte et al, 2002) measures change in both participants' relationship to one another.'	Do planned outcome measures show sensitivity to change pre-/post-/3-months post BCPPA?<br><br>What are the barriers and facilitators to implementation of BCPPA in an NHS setting?<br><br>What is the optimal sample size for a future effectiveness study?<br><br>What is the most appropriate analysis and method to measure costs and outcomes in a future trial?
5.	Of a more technical note:<br>Heterogeneity of the Patient Population<br>The inclusion criteria appear broad, covering different dementia types (e.g., PPA, FTD, AD), distinct symptom profiles and progression patterns. Further justification for combining these populations is needed.	Whilst the patient population is heterogenous we know that dementia symptoms impact conversations, and BCPPA is a tailored intervention that addresses conversation irrespective of linguistic or cognitive communication difficulties. The following has been added to the introduction to make this clearer:<br><br>"Communication difficulties across all dementia types result in a breakdown in conversational exchanges, whether due to word finding, sentence formulation or more general cognitive impairments (Kindell et al, 2017). "<br><br>And<br><br>"Independently of individual linguistic or cognitive communication difficulties, CPT draws on retained strengths, such as gesture, to maintain interactional flow"
6.	It would also be important to clarify whether disease severity will be recorded (e.g., cognitive impairment level, CDR score), and whether this will be accounted for in the analysis.	Given the heterogeneity of the group and the focus on communication in this study we plan to collect data for included and excluded participants on:<br>diagnosis,<br>time since symptom onset<br>time since diagnosis.<br><br>We will also collect data for all included participants on severity of receptive and expressive language and communication

	skills (discourse, conversation) at each stage of study which will clarify disease trajectory and severity of communication skills. We hope this will provide us with comparable communication severity data across conditions.<br><br>This will be taken into account at the analysis stage both in exploration of recruitment and retention data and in terms of outcomes.
7. Sample Size and analysis plan<br>The proposed sample size (n=36) appears small relative to the breadth of the research questions, particularly given the plan of quantitative approach.. The study aims to address multiple dimensions (implementation, feasibility, and future cost-effectiveness considerations), which will likely require subgroup analyses.<br>Given the heterogeneity in both patient population (e.g., dementia subtype) and intervention delivery (e.g., dosage and schedule of BCPPA), further stratification may substantially reduce statistical power and increase the risk of selection bias. The use of repeated measures ANOVA (with group as a between-subject factor and time as a within-subject factor) may not be robust in the context of small sample sizes and potential missing data. Prior literature suggests that substantially larger samples (~114 participants) may be required for adequate statistical power in similar designs (Osstyn, S. L., 2026).<br>If feasible, the authors may consider increasing the sample size, or narrowing this particular study's aims (e.g., focusing more explicitly on feasibility or incorporating a stronger qualitative component)	Many thanks for highlighting the relatively small sample size.<br>Importantly, participants and SLTs delivering the intervention in the previous face to face pilot reported that the measures did not accurately represent the full benefits of the intervention. This study is therefore informed by recent core outcome sets for PPA that highlighted the importance of “having conversations with families and friends” as a priority outcome – for which we are piloting new measures and for which we currently lack a sample size calculation. Therefore the effect size calculation planned in our pilot study protocol will inform a future sample size calculation for an effectiveness study. This has been made clearer with the addition of the following research question:<br><br>“What is the optimal sample size for a future effectiveness study?”
8. Outcome Measures<br>While the selected outcomes are appropriate, the progressive nature of dementia raises questions about the expected duration of effects. The authors may consider whether long-term outcomes will be expected and if that should be assessed.	Indeed, we plan to review maintenance of outcomes 3-months post intervention. We have highlighted this more clearly in the research questions:<br><br>“Do planned outcome measures show sensitivity to change pre-/post-/3-months post BCPPA?”<br><br>It would be good to explore over longer periods in future trials.

9. In addition, given the dyadic nature of the intervention and the stated aim to explore perspectives relevant to future economic evaluation, the inclusion of caregiver-reported outcomes (e.g., subjective burden, quality of life) should be considered.<br>Osstyn, S. L., Handels, R., Rupprecht, C., Boots, L. M. M., & de Vugt, M. E. (2026). Cost-consequence analysis of the partner in balance program for informal carers of persons with dementia. Aging & Mental Health, 30(3), 553–562. https://doi.org/10.1080/13607863.2025.2573845	Many thanks for this comment and this will certainly be considered in design of a future trial. As outlined in tables 1 and 2 care partners will be completing the following caregiver reported outcomes - the EQ5D, the ICECAP-O and the quality of caregiver patient relationship (QCPR). We have also identified several measure to meet the core outcome set items for research in this area. The purpose of this pilot is to refine/reduce the number of listed outcome measures and that would allow inclusion of further measures in future trials.

VERSION 2 - REVIEW

Reviewer 1

Name Buskens, Erik

Affiliation University of Groningen, University Medical Center Groningen, Epidemiology

Date 26-Jun-2026

COI

The authors have addressed the issues raised in relation to this protocol paper

VERSION 2 - AUTHOR RESPONSE

Reviewer 1

The authors have addressed the issues raised in relation to this protocol paper

Many thanks for the positive feedback
