# Supplementary material for: ‘Better Conversations with Primary Progressive Aphasia and other rare dementias (BCPPA plus)’ telehealth communication partner training programme: protocol for an NHS embedded randomised controlled feasibility and implementation study
Source: BMJ Open. 2026 Aug 7;16(8):e117509. doi: 10.1136/bmjopen-2026-117509 (PMC13475093; doi:10.1136/bmjopen-2026-117509)
Supplement: Supplementary file 3 [file bmjopen-16-8-s001.docx]

**TIDieR (Template for Intervention Description and Replication) Checklist for Better Conversations**

| Item Number | Item |
| --- | --- |
| 1. | **Brief name**  Better Conversations (BC) |
| 2. | **Why**  BC is a manualised communication partner training intervention. The goal of BC is to improve communication between people with communication difficulties (such as stroke related aphasia, primary progressive aphasia and Parkinson’s) and their conversation partners (CPs), who are both involved in the training. This intervention was initially developed for people with stroke aphasia (Better Conversations with Aphasia - BCA) and has been refined to meet the needs of people with Primary Progressive Aphasia (Better Conversations with PPA - BCPPA) and Parkinsons (Better Conversations with Parkinsons - BCP). The rationale is that by working with people with communication difficulties and their partners, use of jointly negotiated communication strategies will be improved and maintained. BC is based on applied Conversation Analysis and behaviour change theory [1]. BC provides multiple opportunities for people to reflect on and practice strategies in therapy sessions, using video feedback to support participants to reflect on the impact of their communication on one another, with a focus on elimination of barriers as well as implementation of facilitator strategies. BC emphasises the joint setting of goals during therapy, facilitated by the speech and language therapist (SLT), thus implementing principles of self-management and self-efficacy [2] to set achievable and personally relevant goals. |
| 3. | **What**  Materials:  The BC programs are accompanied by session plans, to guide the SLT, outlining specific goals and activities for the sessions. Participants pre-intervention video-recorded conversation samples are used to provide clips for video feedback during intervention sessions. Handouts and homework sheets have been designed to accompany each session. The following provides a list of example handouts and homework sheets used in the sessions:   - Handout 1: How does conversation work? - Handout 2: What can go wrong in conversations? - Session 1 Homebased task 1: Your conversation troubles and repairs - Handout 3: Goal setting - Session 2 & 3 Home based task 1: A chance to practice some strategies - Handout 5: Information for the future - Certificate of completion (optional)   The BC team provide training to researchers and SLT collaborators in all their studies. The BC Lab offers the training externally twice annually. Some of the BC resources are hosted on the UCLeXtend website at <https://extend.ucl.ac.uk/> and on the BC Lab website and are publicly accessible. For the purposes of the current BC will be delivered over 4-6 one-hour sessions using the described handouts. |
| 4. | **What**  Procedures: The person with the communication difficulties and their CP (a dyad) are provided with information and education on conversation (what it is and how it works). The dyad is then supported to reflect on video samples of their own conversation to identify behaviours that facilitate or are a barrier to communication. Consequently participants are supported to set goals using the Goal Attainment Scales [3] to identify target communication strategies which they practise during activities, role play and homework tasks. Finally, the dyad is supported to plan for future communication needs such as sharing strategies with friends. |
| 5. | **Who provided**  In the BC research studies BC is delivered by qualified speech and language therapists, who have been trained using the online training modules (<https://extend.ucl.ac.uk/>) , a bespoke one-day BC training course (add website url) and regular clinical supervision from speech and language therapists experienced in delivering CPT. |
| 6. | **How**  BC is delivered face to face or online via video conferencing to a person with communication difficulties and their chosen CP. BC may also be delivered in group sessions with other dyads present. Whether online or in person we typically recommend no more than 4-5 dyads in a BC group. For the purposes of the current study BC will be delivered only individually to a person with a communication difficulty and their chosen CP online via video conferencing (on zoom). |
| 7. | **Where**  BC can be delivered at home or in an outpatient type setting, depending on the location of speech and language therapy provision. It can also be delivered remote via video conferencing. For these purposes of the current study BC will be delivered video conferencing (on zoom). |
| 8. | **When and how much**  The BC programs are delivered based on the requirements of the dyad and how long it takes them to achieve their goals. However, constraints such as the available services may influence this decision. Typically, individual BC programs are provided once weekly for around 60 minutes. Group BC programs may be delivered over shorter and more intensive time frames. Individual BC intervention research will be provided detailed information about number and duration of sessions, schedule and frequency. For the purposes of this study, sessions were delivered approximately once per week over 4-6 sessions. |
| 9. | **Tailoring**  BC is tailored to each individual dyad (a person with communication difficulties and their CP) by using video samples of their conversation recorded before intervention. These samples permit the SLT to assess the dyad’s communication difficulties and strengths before commencing the program. They are also used for video feedback to allow a dyad to set goals i.e. target communication strategies, which they practise during activities, role play and home based tasks. |
| 10. | **Modifications**  BC interventions are being modified for each intervention study. Each reported study should provide a detailed explanation of modifications undertaken. For the purposes of the current study the BCPPA patient and public involvement group co-refined the intervention, which including the following modifications   - Technical support materials –easy to use youtube video tutorials (https://youtu.be/vLS6gbLSXAg) and one-page handouts were produced to aid recording of conversations and attendance at video conferencing meetings. - Handouts were refined – handouts were made significant shorter and tailored to address communication difficulties across rare dementias - Increased flexibility in the number of sessions: The four stages of the BCPPA intervention can delivered over four to six sessions as jointly decided by the person with PPA, their CP and the speech and language therapist. - Introduction of a reward- A certificate is provided as an optional reward for participants - Recommendation for practice topics to discuss for home-based tasks - Participants are supported to identify how to make practice rewarding and routine to promote generalisation post therapy |
| 11. | **How well**  All BC intervention sessions are video recorded for fidelity assessment and a detailed protocol has been developed to support the BC program of research [4]. |

1. Johnson FM, Best W, Beckley FC, Maxim J, Beeke S. Identifying mechanisms of change in a conversation therapy for aphasia using behaviour change theory and qualitative methods. Int J Lang Commun Disord. 2017;52:374–87. doi:10.1111/1460-6984.12279.

2. Yorkston K, Baylor C, Britton D. Incorporating the Principles of Self- Management into Treatment of Dysarthria Associated with Parkinson ’ s Disease. Semin Speech Lang. 2017;1:210–9.

3. Turner-Stokes L. Goal attainment scaling (GAS) in rehabilitation: a practical guide. Clin Rehabil. 2009;23:362–70. doi:10.1177/0269215508101742.

4. Volkmer, A., Beeke, S., Warren, J. D., Spector, A., & Walton, H. (2024). Development of fidelity of delivery and enactment measures for interventions in communication disorders. *British Journal of Health Psychology*, *29*(1), 112-133.
