# Supplementary material for: ‘Better Conversations with Primary Progressive Aphasia and other rare dementias (BCPPA plus)’ telehealth communication partner training programme: protocol for an NHS embedded randomised controlled feasibility and implementation study
Source: BMJ Open. 2026 Aug 7;16(8):e117509. doi: 10.1136/bmjopen-2026-117509 (PMC13475093; doi:10.1136/bmjopen-2026-117509)
Supplement: Supplementary file 4 [file bmjopen-16-8-s002.docx]

**Dynamic assessment of telehealth learning**

Initials: ______________	Date: ____________

***Instructions for administration:***

*Go through the different prompts, starting with verbal instructions for part 1, once complete say the verbal instructions for part 2. Each time you give a prompt, tick the relevant box. Add up the number of ticks for each attempt at the task - this is the prompt score for that attempt (maximum 51 points)*

*Repeat the task x3 but discontinue if the person is able to perform the task correctly with only prompt #1 (verbal instructions for part 1 and 2, completing the whole task).*

*Add the prompt score from the 3 attempts to give a total prompt score for that task (maximum 153)*

*To obtain a learning score, subtract the prompt score on the final attempt from the prompt score on the first attempt (e.g. T1: 15/51, T3: 5/51= learning score of 10)*

*Time each attempt, starting from after you’ve delivered the verbal instructions for the whole task and finishing when the participant has completed the task.*

***Telehealth task:***

***Before starting:***

- ***Ensure that there is an e-mail program and Zoom installed on the device***
- ***The laptop or iPad should be locked or ‘sleeping’***

Verbal instructions only for part 1 (4 steps):

**“Switch on the laptop and open your e-mail. You will see a message with a Zoom link. Open the link then press allow”**

Verbal instructions only for part 2 (4 steps):

**“Join computer audio. Turn your camera on. Turn you microphone off and on. Close the Zoom program.**

**Telehealth task: 1^st^ attempt**

| **1. Verbal instructions (whole task)** |  | **2. Verbal instructions (step-by-step)** | **3. Written instructions with picture prompts** | | **4. Prompt with pointing and/or gesture** | **5. Demonstrate** | **6. Hand over hand facilitation** |
| --- | --- | --- | --- | --- | --- | --- | --- |
| Part 1 | Unlock the laptop or iPad |  |  | |  |  |  |
|  | Open an e-mail program |  |  | |  |  |  |
|  | Click on the Zoom link in the message |  |  | |  |  |  |
|  | Press ‘allow’ |  |  | |  |  |  |
| Part 2 | Press ‘join with computer audio’ |  |  | |  |  |  |
|  | Turn your camera on |  |  | |  |  |  |
|  | Turn your microphone off and on |  |  | |  |  |  |
|  | Close the Zoom program |  |  | |  |  |  |
| Prompt score= /51 | | | | Time taken= | | | |

**Telehealth task: 2^nd^ attempt**

| **1. Verbal instructions (whole task)** |  | **2. Verbal instructions (step-by-step)** | **3. Written instructions with picture prompts** | | **4. Prompt with pointing and/or gesture** | **5. Demonstrate** | **6. Hand over hand facilitation** |
| --- | --- | --- | --- | --- | --- | --- | --- |
| Part 1 | Unlock the laptop or iPad |  |  | |  |  |  |
|  | Open an e-mail program |  |  | |  |  |  |
|  | Click on the Zoom link in the message |  |  | |  |  |  |
|  | Press ‘allow’ |  |  | |  |  |  |
| Part 2 | Press ‘join with computer audio’ |  |  | |  |  |  |
|  | Turn your camera on |  |  | |  |  |  |
|  | Turn your microphone off and on |  |  | |  |  |  |
|  | Close the Zoom program |  |  | |  |  |  |
| Prompt score= /51 | | | | Time taken= | | | |

**Telehealth task: 3^rd^ attempt**

| **1. Verbal instructions (whole task)** |  | **2. Verbal instructions (step-by-step)** | **3. Written instructions with picture prompts** | | **4. Prompt with pointing and/or gesture** | **5. Demonstrate** | **6. Hand over hand facilitation** |
| --- | --- | --- | --- | --- | --- | --- | --- |
| Part 1 | Unlock the laptop or iPad |  |  | |  |  |  |
|  | Open an e-mail program |  |  | |  |  |  |
|  | Click on the Zoom link in the message |  |  | |  |  |  |
|  | Press ‘allow’ |  |  | |  |  |  |
| Part 2 | Press ‘join with computer audio’ |  |  | |  |  |  |
|  | Turn your camera on |  |  | |  |  |  |
|  | Turn your microphone off and on |  |  | |  |  |  |
|  | Close the Zoom program |  |  | |  |  |  |
| Prompt score= /51 | | | | Time taken= | | | |

**Total prompt score** (add scores for attempts 1, 2 and 3) =	/153

**Learning score**: difference between first and final attempt at PC task =

Qualitative observations:

Does participant know what they need to do, but has motor, sensory or cognitive difficulties carrying out task?

If the participant finds the task difficult, how do they react? (e.g. level of frustration, are they keen to try again/practise/problem solve?)

Which prompts does the participant respond best to?

Is the participant able to reflect on how difficult the tasks were, and which prompts they found helpful?

Any other comments?
